# Redescriptions of *Spinitectus acipenseri* and *S*. *micracanthus* (Nematoda, Cystidicolidae), with notes on the taxonomy of *Spinitectus*-like nematodes parasitising North American fishes[Fn FN1]

**DOI:** 10.1051/parasite/2023036

**Published:** 2023-09-07

**Authors:** František Moravec, David G. Huffman, Isaure de Buron, David González-Solís

**Affiliations:** 1 Institute of Parasitology, Biology Centre of the Czech Academy of Sciences 37005 České Budějovice Czech Republic; 2 Freeman Aquatic Biology, Texas State University-San Marcos San Marcos Texas 78666-4616 USA; 3 Department of Biology, College of Charleston Charleston South Carolina 29424-0001 USA; 4 El Colegio de la Frontera Sur (ECOSUR), Unidad Chetumal Chetumal Quintana Roo 77049 Mexico

**Keywords:** Parasitic nematode, *Ctenascarophis*, *Prospinitectus*, Morphology, Canada, Texas, South Carolina, USA

## Abstract

Based on light microscopical and scanning electron microscopical (SEM) examinations, two North American species of *Spinitectus* Fourment, 1884, *S*. *acipenseri* Choudhury & Dick, 1992 and *S*. *micracanthus* Christian, 1972 (Nematoda, Cystidicolidae) are redescribed from museum voucher specimens (*S*. *acipenseri*) and those newly collected from centrarchid and some other fishes in the Upper San Marcos River in Texas and the Santee River in South Carolina, USA. The first use of SEM to study *S*. *acipenseri*, a parasite of lake sturgeon *Acipenser fulvescens* Rafinesque (Acipenseridae) in Canada, made it possible to describe dorsal and ventral lips, amphids and sublabia, and the presence of a dorsal barb on the right spicule, which was confirmed to be the most characteristic feature of this species. The SEM study of *S*. *micracanthus*, a parasite mainly of centrarchids, enabled us to correctly determine the location of the excretory pore in relation to rings of cuticular spines in the male, and to describe the exact structure of the tip of the male tail, sublabia, phasmids and the presence of a median ventral protuberance on the male tail. Some taxonomic problems of North American species of *Spinitectus* are discussed. *Filaria serrata* Linton, 1901 is considered a junior synonym of *S*. *oviflagellis* Fourment, 1884. To date, there are 13 valid species of *Spinitectus* parasitising fishes in North America. Keys to species of *Spinitectus*-like nematodes from fishes in North American waters are provided.

## Abstract (fr)

Deux espèces nord-américaines de *Spinitectus* Fourment, 1884, *S. acipenseri* Choudhury & Dick, 1992 et *S. micracanthus* Christian, 1972 (Nematoda, Cystidicolidae) sont redécrites à partir de spécimens de musée et d’autres nouvellement collectés aux USA dans les rivières Upper San Marcos (Texas) and Santee (Caroline du Sud), avec l’aide de la microscopie optique et électronique à balayage (MEB). L’utilisation pour la première fois du MEB pour étudier *S*. *acipenseri*, un parasite de l’esturgeon *Acipenser fulvescens* Rafinesque (Acipenseridae) au Canada, a permis de décrire les lèvres dorsales and ventrales, les amphides et sublabia, ainsi que la barbe dorsale sur le spicule droit que nous confirmons être le critère le plus caractéristique de cette espèce. L’étude au MEB de *S. micracanthus*, un parasite principalement de centrarchides, nous a permis de déterminer correctement la place du pore excréteur en relation avec les couronnes d’épines cuticulaires chez le mâle, ainsi que de décrire la structure exacte de l’extrémité caudale du mâle, des sublabia, des phasmides et la présence d’une protubérance médiane et ventrale sur la queue du mâle. Des problèmes taxonomiques sur les espèces nord-américaines de *Spinitectus* sont discutés. *Filaria serrata* Linton, 1901 est considéré synonyme plus récent de *S*. *oviflagellis* Fourment, 1884. À ce jour, il y a 13 espèces valides de *Spinitectus* parasites de poissons en Amérique du Nord. Des clés des espèces des nématodes de type *Spinitectus* provenant des poissons des eaux nord-américaines sont fournies.

## Introduction

The nematode genus *Spinitectus* Fourment, 1884 (Cystidicolidae, Habronematoidea) includes a large number of species described mainly from the digestive tract of freshwater and marine fishes throughout the world [37]. Representatives of this genus, as well as a few species of the related genera *Ctenascarophis* Mamaev, 1968 and *Prospinitectus* Petter, 1979, are conspicuous in that their body surface bears numerous transverse rings or rows of cuticular spines. Even though the number of spines per specific ring may exhibit a high degree of intraspecific variability in some species of *Spinitectus* (*e.g.*, [30, 41]), the size, number and distribution of cuticular spines are generally considered to be very important taxonomic features in these nematodes.

Another noteworthy characteristic of *Spinitectus* spp. is the external appearance of the fully developed egg, which may be smooth or covered with a very thin, indistinct gelatinous coating, or the eggs may possess polar filaments or caps or conspicuous equatorial swellings or globules (sometimes called “floads”) [32]; in this regard, eggs of *Spinitectus* spp. sometimes resemble those of the genus *Rhabdochona* Railliet, 1916 (Rhabdochonidae, Thelazioidea), widely distributed parasites of freshwater fishes [33]. Unique among all cystidicolids are the recently described eggs of *Spinitectus mirabilis* Moravec & Nagasawa, 2021, a parasite of the freshwater fish *Kuhlia rupestris* (Lacepède) (Kuhliidae) in Okinawa Prefecture, Japan, where each egg is provided with both polar filaments and lateral swellings; however, in contrast to other *Spinitectus* spp. or those of *Rhabdochona* spp., the lateral swellings of *S*. *mirabilis* are more elongate, with a lobular surface, thus resembling the lateral swellings (reported also as mammillations) on the eggs of *Cystidicola stigmatura* (Leidy, 1886), a swimbladder nematode parasite of Nearctic salmonids [40].

Despite the abundance of countable and measurable external appendages on adult *Spinitectus* nematodes, the identification of these worms to species is surprisingly difficult because the existing descriptions of most species were based on superficial study by bright-field light microscopy (LM) and are inadequate. The small body size renders LM to be an inadequate tool for properly diagnosing the intricate anatomy of cephalic and cuticular structures, deirids, precloacal ridges, and posterior-most pairs of male genital papillae or phasmids. Therefore, the use of scanning electron microscopy (SEM) is herein recommended as minimally necessary for the proper assessment of these important diagnostic structures [32].

From the physico-geographical point of view, as used herein, North America includes not only Northern America, but also Central America, islands of the American Mediterranean Sea (= the Caribbean Sea and the Gulf of Mexico) and Greenland, representing thus the largest continent of the Western Hemisphere. The current literature regarding species of *Spinitectus* and other *Spinitectus*-like nematodes occurring in this vast area is unsatisfactory, especially because of inadequate attention to certain anatomical details in descriptions, and some taxonomic problems resulting from reports of *Spinitectus* from freshwater fishes in the USA and Canada. Apparently, frequent mis-assignment of specimens to species within the genus, particularly in faunistic surveys, may have been caused by failure of various authors to recognise that some collections under study had consisted only of subadults, and that morphologically relevant distinctions between collections from obligate definitive hosts *versus* those from facultative hosts (such as paratenic, paradefinitive or postcyclic hosts [44]) had not been taken into consideration.

Recent LM and SEM examinations of *Spinitectus* specimens from freshwater fishes of the USA and Canada provided opportunities to redescribe two species in much greater detail and to also elucidate some prevalent taxonomic problems in the literature associated with North American representatives of the genus. The results of this study are presented below.

## Materials and methods

The specimens of *Spinitectus* studied were drawn from the following three collections:

1. Ethanol-preserved voucher specimens of *S*. *acipenseri* Choudhury & Dick, 1993 from lake sturgeon *Acipenser fulvescens* Rafinesque, Canada, deposited in the Helminthological Collection of the Institute of Parasitology, Biology Centre of the Czech Academy of Sciences, České Budějovice, Czech Republic (IPCAS N-659) (donated by Anindo Choudhury in 1993).
2. Specimens of *Spinitectus* (adults and larvae) from sunfishes *Lepomis auritus* (Linnaeus), *L*. *cyanellus* Rafinesque, *L*. *macrochirus* Rafinesque, *L*. *punctatus* (Valenciennes), *Lepomis* sp. (hybrids) and largemouth black bass *Micropterus salmoides* (Lacepède) (all Centrarchidae), and larval *Spinitectus* also from Mexican tetra *Astyanax mexicanus* (De Filippi) (Characidae) and largespring gambusia *Gambusia geiseri* Hubbs & Hubbs (Poeciliidae), collected by the authors (D.G. Huffman and F. Moravec) from the Upper San Marcos River (29.889663, −97.934373) in San Marcos, Texas, USA in May 1987 and September 1999.
3. Specimens of *Spinitectus* (adults and larvae) from *L*. *macrochirus* and *M*. *salmoides* (both Centrarchidae) collected by the authors (I. de Buron, D. González-Solís and F. Moravec) from the Santee River (33.449231, −80.161669 to 33.403057, −79.854879), South Carolina, USA in April 2007.

Examined fish were caught by angling and seining in Texas and electrofishing in South Carolina. The digestive tract was examined, and nematodes collected were washed in physiological saline and then fixed and preserved in 4% formalin. For LM examination, the nematodes were cleared with glycerine. Drawings were made with the aid of a Zeiss drawing attachment. Specimens used for SEM were postfixed in 1% osmium tetroxide (in phosphate buffer), dehydrated through a graded acetone series, critical-point-dried and sputter-coated with gold; they were examined using a JEOL JSM-6300 scanning electron microscope at an accelerating voltage of 15 kV in 1993 (Figs. 2 and 4), or a JEOL JSM-7401F scanning electron microscope at an accelerating voltage of 4 kV, GB low mode in 2022 (Figs. 5–7). All measurements in species descriptions are in micrometres unless otherwise indicated. Voucher specimens were deposited in the Helminthological Collection of the Institute of Parasitology, Biology Centre of the Czech Academy of Sciences, České Budějovice, Czech Republic (IPCAS). The fish nomenclature follows FishBase [16].

## Results

Family Cystidicolidae Skryabin, 1946

### *Spinitectus acipenseri* Choudhury & Dick, 1992 Figures 1, 2

Host: *Acipenser fulvescens* Rafinesque (Acipenseridae, Acipenseriformes).

**Figure 1:**
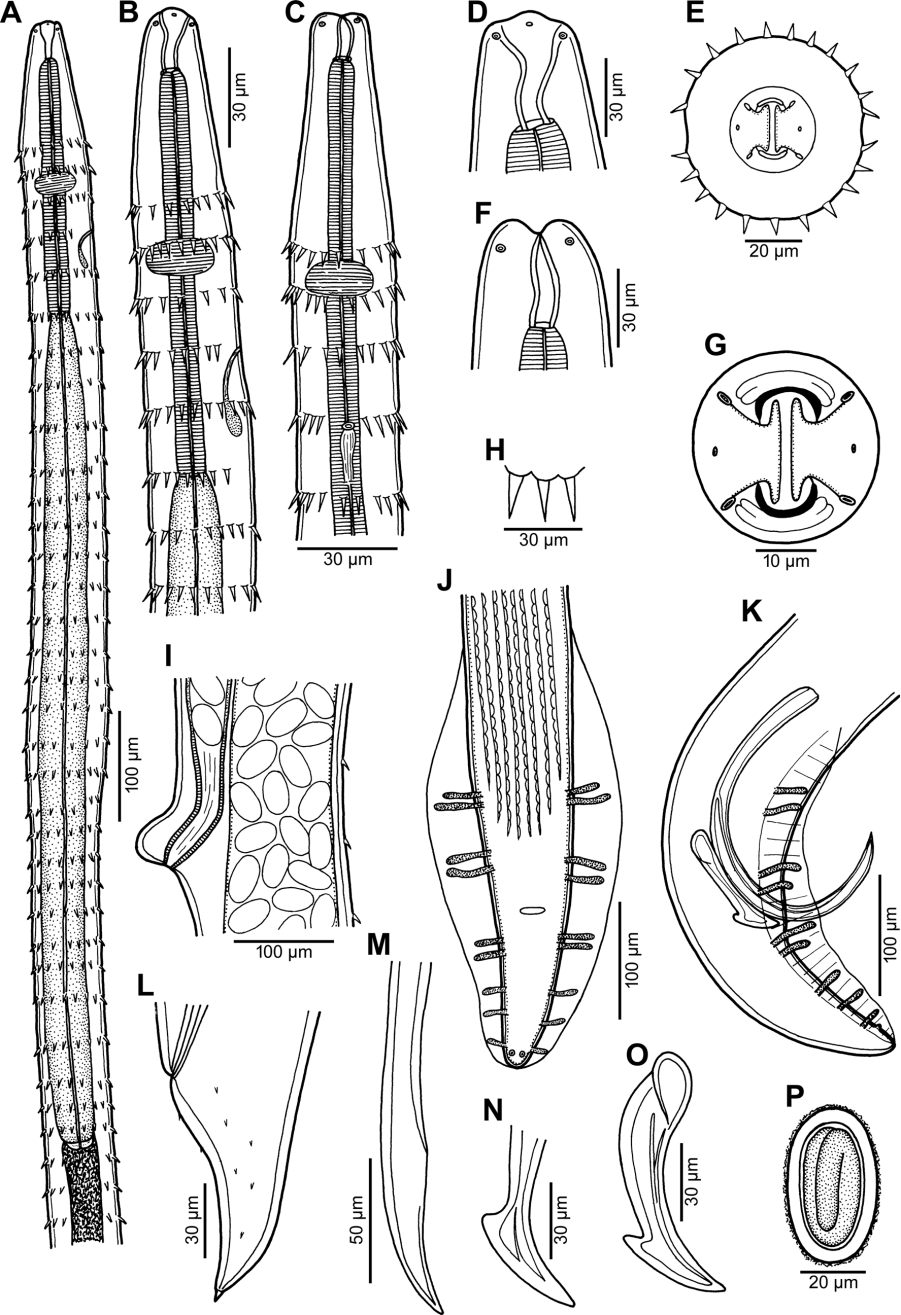
*Spinitectus acipenseri* Choudhury & Dick, 1992 from *Acipenser fulvescens* of Canada. (A) Oesophageal part of male body, lateral view; (B, C) anterior end of male, lateral and ventral views, respectively; (D–F) cephalic end of male, lateral, apical and dorsoventral views, respectively; (G) mouth, apical view; (H) shape of spines at second transverse row, lateral view; (I) region of vulva, lateral view; (J, K) posterior end of male, ventral and lateral views, respectively; (L) tail of female, lateral view; (M, N) distal tips of left and right spicule, respectively, lateral views; (O) right spicule, lateral view; (P) mature egg.

**Figure 2:**
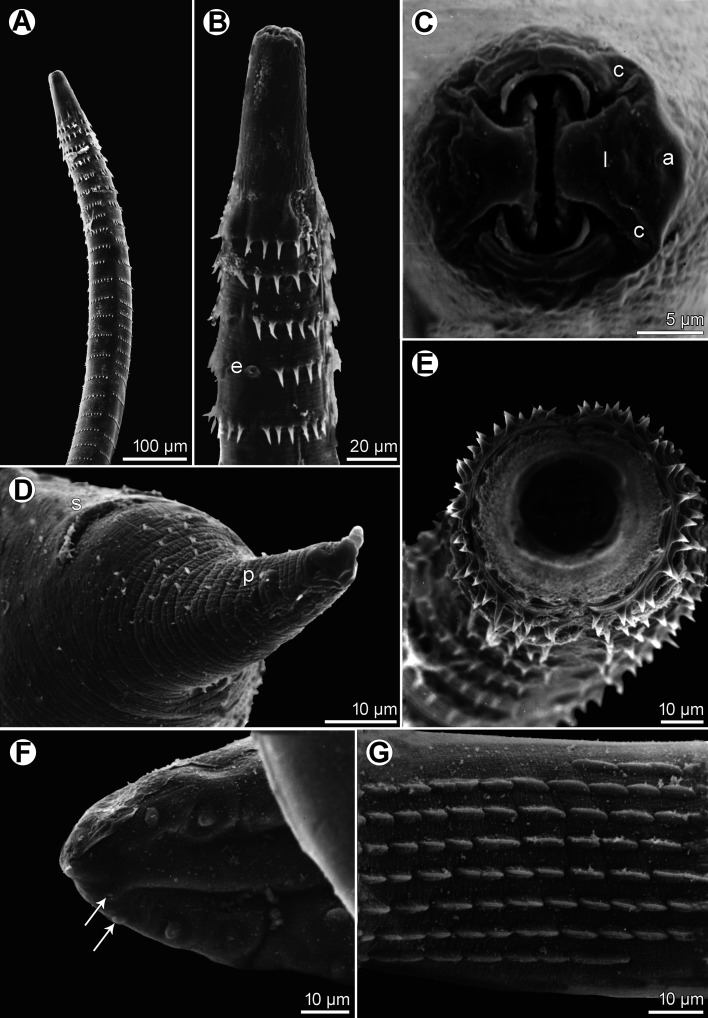
*Spinitectus acipenseri* Choudhury & Dick, 1992 from *Acipenser fulvescens* of Canada, scanning electron micrographs. (A) Anterior part of body, lateral view; (B) anterior end, ventral view; (C) cephalic end, apical view; (D) female tail, ventrolateral view; (E) anterior end of body, apical view (focused on anterior rows of cuticular spines); (F) tail tip of male, ventral view (arrows indicate postanal papillae of two posteriormost pairs); (G) ventral precloacal tesellated cuticular ridges of male, ventral view. (a) amphid; (c) cephalic papilla; (e) excretory pore; (l) pseudolabium; (p) phasmid; (s) anus.

Site of infection: Stomach.

Locality: Cumberland Lake (Saskatchewan River), Canada (collected in June 1989).

Deposition of voucher specimens: IPCAS N-659.

#### Description

*General*: Small, whitish nematodes. Body elongate; cephalic end blunt, posterior end conical, with pointed tip. Surface of body with transverse rings of posteriorly directed cuticular spines (Figs. 1A–1C, 1H, 2A, 2B, 2E); rings interrupted on both sides of body by narrow longitudinal lanes barren of spines; some more posterior rings incomplete or single spines present. Longitudinal spacing of first 2 rings of spines tighter than subsequent rings; more posterior rings almost equally spaced (Figs. 1A–(1C, 2A–2C). First ring with 20–22 spines; longest spines occur in 2nd and 3rd rings. Larger spines in *ca.* 8 anterior rings, then spines gradually diminish in length posteriad (Figs. 1A, 2A). In males, spines absent from about posterior third of body; in females, small individual randomly arranged spines continue posteriad nearly to tail tip (Figs. 1L, 2D). Oral aperture oval, dorsoventrally elongated, bounded dorsally and ventrally by low, large C-shaped labia and backed-up with stout bases (Figs. 1G, 2C) that form dorsal and ventral margins of oral opening. One simple, narrow, bent sclerotised structure (sublabium), with a somewhat thickened free margin, attached by its base to inner surface of each labium. Lateral pseudolabia large, almost occluding mouth. In apical view, thin, opposed, plate-like inner parts of pseudolabia extend dorsoventrally, apparently forming occlusive surfaces of molar-like pseudolabia. Lateral bases of pseudolabia much wider dorsoventrally than occlusive part, with single cephalic papilla at dorsal and ventral ends of pseudolabial bases (for total of 4 arranged as corners of a square); margins of both pseudolabia straight, aligned dorsoventrally, parallel to each other. One amphid situated at mid-lateral edge of pseudolabial base (Figs. 1E, 1G, 2C). Vestibule very short, ending 1/3–1/8 anterior to 1st ring of spines, with distinct anterior prostom in lateral view (Figs. 1A–1D, 1F). Deirids not found. Oesophagus divided into anterior muscular and much longer, somewhat wider posterior portion (Figs. 1A–1C); length ratio of portions 1:3.1–3.9. Nerve ring encircles muscular oesophagus at level of 1st and 3rd rings of spines. Excretory pore situated between 3rd and 4th rings of spines in male, and at level of 4th ring in female (Figs. 1A, 1B, 2B). Males smaller than gravid females.

*Male* (2 specimens): Length of body 4.24–5.44 mm, maximum width 82–95. First ring of spines 129–150 from anterior extremity; spines in this ring 9–12 long. Maximum length of spines 12–15 in 2nd and 3rd rings. Length of vestibule including prostom 33–48; prostom in smaller specimen 12 long, 21 wide. Muscular oesophagus 264–345 long, 18 wide; glandular oesophagus 0.84–1.07 mm long, 51–60 wide; length ratio of both parts of oesophagus 1:3.1–3.2. Length of entire oesophagus and vestibule represents 26% of body length. Nerve ring and excretory pore 162–177 and 222–276, respectively from anterior extremity. Posterior end of body ventrally curved, provided with well-developed vesiculated subventral alae. Ventral precloacal ridges (area rugosa) present, formed by 8 longitudinal rows of tessellated outgrowths (Figs. 1J, 2G). Preanal papillae: 4 pairs of subventral pedunculate papillae, of which 1st and 2nd, and 3rd and 4th pairs are close to each other. Postanal papillae: 5 pairs of subventral pedunculate papillae and 1 pair of small ventral papillae situated posterior to subventrals (Figs. 1A, 1B, 2F). Phasmids not observed. Large (left) spicule narrow, 288–312 long, with pointed tip; length of its shaft in smaller specimen 108 (38% of spicule length). Small (right) spicule narrow, 78–96 long, with large dorsal barb on distal end (Figs. 1K, 1M–1O). Length ratio of spicules 1:3.3–3.7. Tail conical, 108–126 long, with rounded tip (Figs. 1J, 1K, 2F).

*Female* (4 gravid specimens; smallest specimen with immature eggs): Length of body 5.97–8.83 mm, maximum width 82–136. First ring of spines 135–168 from anterior extremity; maximum length of spines 12–15 in 2nd and 3rd rings. Length of vestibule including prostom 33–45; funnel-shaped prostom 12–15 long, 21–24 wide. Muscular oesophagus 282–378 long, maximum width 18–24; glandular oesophagus 0.99–1.29 mm long, 54–72 wide; length ratio of both parts of oesophagus 1:3.3–3.9. Length of entire oesophagus represents 19–23% of body length. Nerve ring and excretory pore 147–210 and 219–294, respectively from anterior extremity. Vulva situated in posterior half of body, 3.92–6.49 mm from anterior extremity (at 66–88% of body length); anterior vulval lip conspicuously elevated. Vagina muscular, short, directed anteriorly from vulva (Fig. 1I). Fully-developed eggs in uterus oval, thick-walled, with fine gelatinous coating on surface, each containing larva (Fig. 1P); size 39–42 × 21–24; thickness of rigid eggshell wall 3. No egg filaments or superficial swellings present. Tail conical, 72–96 long, with small outgrowth 5–6 long at tip; surface of tail with several scattered minute cuticular spines (Figs. 1L, 2D).

#### Remarks

*Spinitectus acipenseri* is one of the two known North American species of the genus (along with *S*. *gracilis*) characterised by the presence of a markedly short vestibule that does not reach posteriad to the mid-point between the anterior extremity and the first ring of cuticular spines. The original description of *S*. *acipenseri* by Choudhury & Dick [7] was based solely on LM studies of specimens from the stomach of the lake sturgeon *A*. *fulvescens* in Saskatchewan and Manitoba, Canada (the only freshwater sturgeon species in North America). The authors distinguished *S*. *acipenseri* from *S*. *gracilis* (a morphologically similar parasite reported from many species of North American fishes) mainly by the larger size and arrangement of spines, total body measurements and the length ratio of oesophagus and body.

The present examination of voucher specimens of *S*. *acipenseri*, especially the first SEM study of the species, made it possible to describe some morphological features in more detail and to reveal other details not previously reported in the original description. Features described herein for the first time are the amphids, dorsal and ventral lips, sublabia and the shaft of the left spicule. However, the main difference between *S*. *acipenseri* and *S*. *gracilis*, *i.e.*, the presence of a conspicuously large dorsal barb on the distal end of the right spicule in *S*. *acipenseri*, is not mentioned as a distinguishing feature in the original Choudhury & Dick [7] description, although this large barb was illustrated on the right spicule in their figs. 6–8.

The life cycle of *S*. *acipenseri* has not yet been reported.

### *Spinitectus micracanthus* Christian, 1972 Figures 3–7

Hosts of studied collections: *Lepomis auritus* (Linnaeus), *L*. *cyanellus* Rafinesque, *L*. *macrochirus* Rafinesque, *L*. *punctatus* (Valenciennes), *Lepomis* (hybrid) and *Micropterus salmoides* (Lacepède) (all Centrarchidae, Centrarchiformes); larvae also in *Astyanax mexicanus* (De Filippi) (Characidae, Characiformes) and *Gambusia geiseri* Hubbs & Hubbs (Poeciliidae, Cypriniformes).

**Figure 3:**
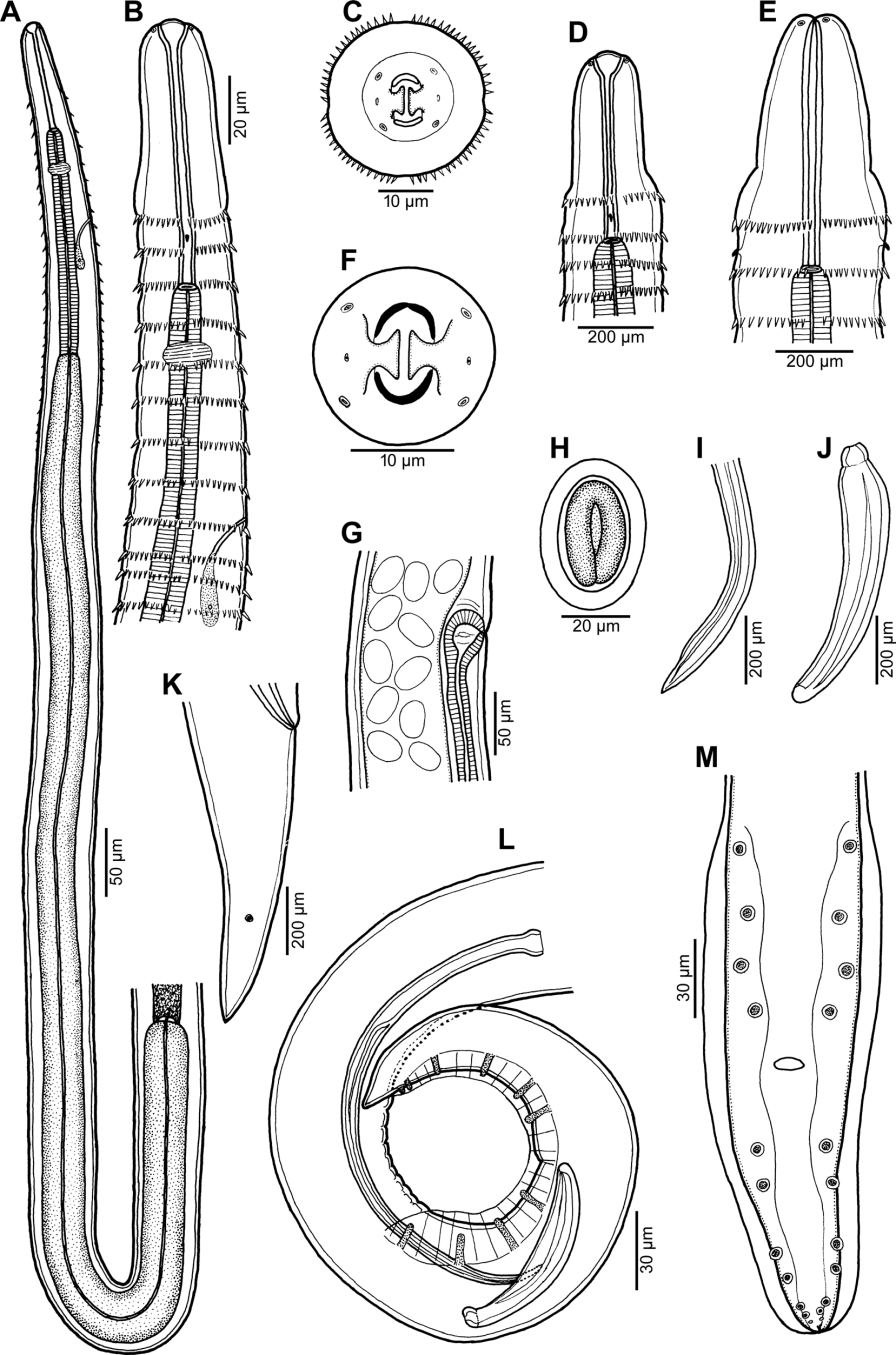
*Spinitectus micracanthus* Christian, 1972 from *Lepomis macrochirus*, Texas. (A) Anterior (oesophageal) portion of male body, lateral view; (B) anterior end of male, lateral view; (C) cephalic end of female, apical view; (D) cephalic end of female, lateral view; (E) cephalic end of male, dorsoventral view; (F) mouth, apical view; (G) region of vulva, lateral view; (H) mature egg; (I) distal end of larger (left) spicule, lateral view; (J) small (right) spicule, lateral view; (K) female tail, lateral view; (L, M) posterior end of male, lateral and ventral views, respectively. (A–D and F–M specimens from *Lepomis macrochirus*; E specimen from *Micropterus salmoides*).

Site of infection: Intestine.

Localities: Upper San Marcos River in San Marcos, Texas, USA (collected in May 1987 and September 1999) and Santee River, South Carolina, USA (collected in April 2007).

Prevalence and intensity: San Marcos River (May 1987): *L*. *auritus*: 3 fish infected/6 fish examined; intensity 1–3 (mean 2) nematodes. *L*. *cyanellus*: 3/8; 2–35 (12). *L*. *macrochirus*: 100% (14/14); 3–40 (14). *L*. *punctatus*: 85% (11/13); 2–35 (6). *Lepomis* (hybrid): 3/3; 3 (3). *M*. *salmoides*: 1/1; 12. *A*. *mexicanus*: 1/2; 1. *G*. *geiseri*: 1/4; 1. Upper San Marcos River (September 1999): *L*. *auritus*: 69% (11/16); 1–12 (4). *L*. *cyanellus*: 3/4; 5–10 (8). *L*. *macrochirus*: 5/9; 2–5 (4). Santee River: *L*. *macrochirus*: 2/4; 1–10 (6). *M*. *salmoides*: 25% (3/12); 1–35 (13).

Deposition of voucher specimens: IPCAS N-262.

#### Description

**Figure 4:**
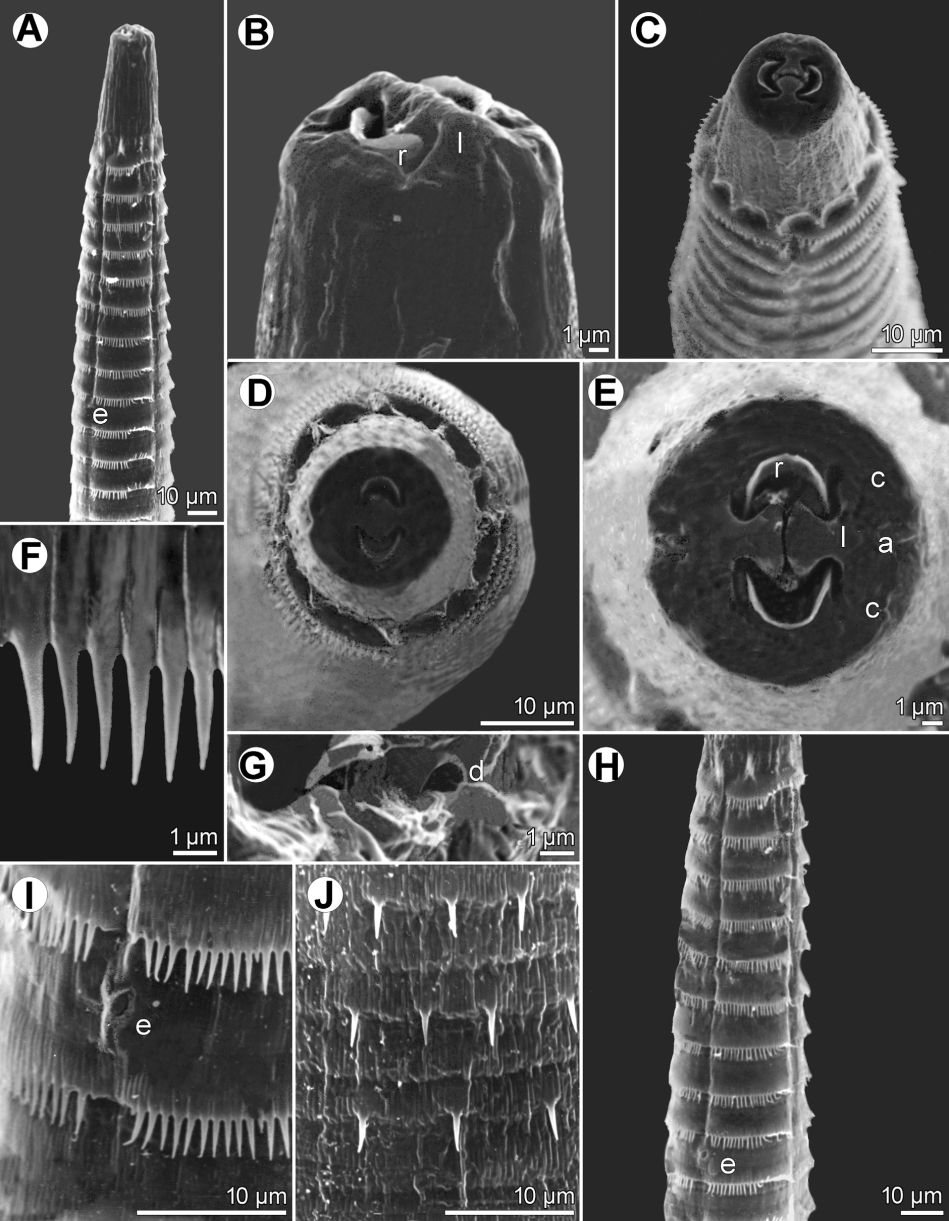
*Spinitectus micracanthus* Christian, 1972 from *Lepomis macrochirus*, Texas, scanning electron micrographs. (A) Anterior end of female, subventral view; (B, C) cephalic end, sublateral and lateral views, respectively; (D) cephalic end, apical view; (E) mouth, apical view; (F) cuticular spines in anterior ring; (G) deirid; (I) excretory pore; (J) cuticular spines in more posterior rings; (H) anterior rings of spines and excretory pore in female, ventral view. (a) amphid; (c) cephalic papilla; (d) deirid; (e) excretory pore; (l) pseudolabium; (r) sublabium.

**Figure 5:**
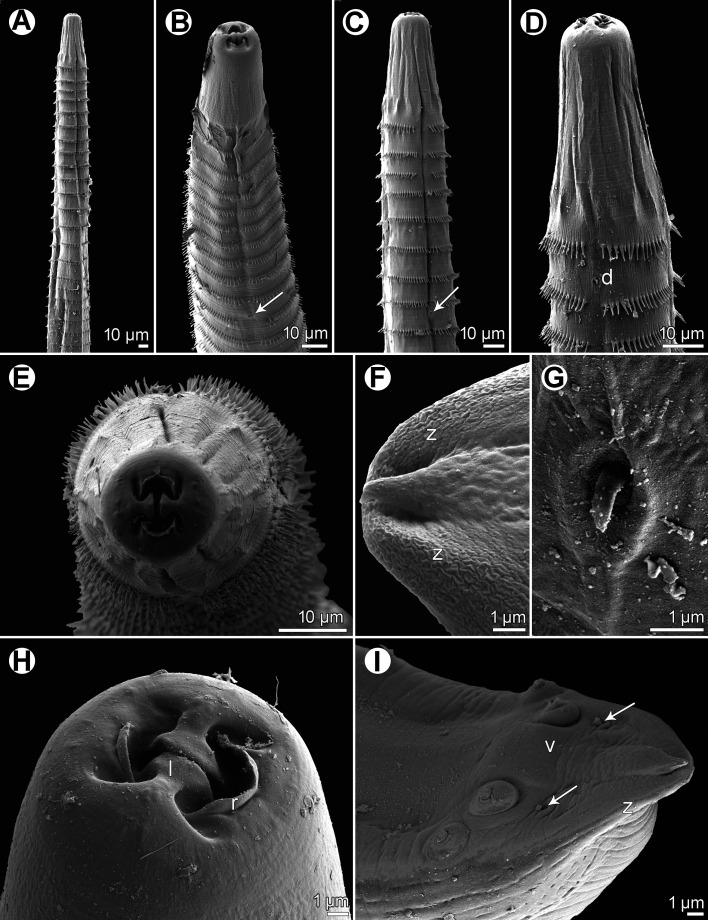
*Spinitectus micracanthus* Christian, 1972 from *Lepomis macrochirus*, Texas, scanning electron micrographs. (A) Anterior end of male, ventral view; (B) anterior end of female, ventral view (arrow indicates excretory pore); (C) anterior end of male, ventral view (arrow indicates excretory pore); (D, E) cephalic end of male, lateral and apical views, respectively; (F) tail tip of male, ventral view; (G) deirid; (H) cephalic end of male, subapical view; (I) posterior end of male tail with two posteriormost pairs of postanal papillae, ventrolateral view (arrows indicate phasmids). (d) deirid; (l) pseudolabium; (r) sublabium; (v) median caudal protuberance; (z) caudal ala.

*General*: Small, whitish nematodes. Body elongate; cephalic end blunt, posterior end conical, with pointed tip. Surface of body with transverse rings of cuticular spines (Figs. 3A–3E, 4A, 4C, 4D, 4F, 4H–4J, 5A–5E, 7A, 7B); rings interrupted by 4 (1 dorsal, 1 ventral and 2 lateral) narrow spineless longitudinal lanes (Figs. 3C, 4A, 4C, 4D, 4I, 4H, 5B–5E, 7A, 7B); some more posterior rings incomplete or single spines present. First two rings of spines not closer to each other than subsequent rings; more posterior rings almost equally spaced, with posteriorly directed spines (Figs. 3B, 3D, 3E, 4A, 4F, 4H, 5A–5D, 7A, 7B). First ring with 69–72 spines; longest spines occur in 3rd–6th rings. Larger spines in *ca.* 12 anterior rings, then spines gradually diminish in length posteriad (Figs. 5A, 7A). Minute spines present on body to about level of anterior part of glandular oesophagus, more posterior part of body smooth in both sexes (Figs. 3A, 7E). Oral aperture oval, dorsoventrally elongated, surrounded by 2 low, large labia, 1 dorsal and 1 ventral, with broad base (Figs. 3C, 3F, 4B–4E, 5E, 5H), forming dorsal and ventral margins of oral opening. One simple, narrow, bent sclerotised structure (sublabium), with a somewhat thickened free margin, attached by its base to inner surface of each labium. Lateral pseudolabia large, almost occluding mouth. In apical view, narrow inner parts of pseudolabia extended dorsoventrally, forming 2 (1 laterodorsal and 1 lateroventral) extensions on each pseudolabium. Inner margins of both pseudolabia straight, aligned dorsoventrally, mutually parallel. Pair of small lateral amphids and 4 small submedian cephalic papillae situated outside pseudolabia (Figs. 3C, 3F, 4B–4E, 5E, 5H). Vestibule narrow, long, reaching posteriorly to level of 2nd–3rd ring of spines, with distinct anterior prostom in lateral view (Figs. 3A, 3B, 3D, 3E). Small simple deirids situated between 1st and 2nd rings of spines (Figs. 3B, 3D, 3E, 4G, 5D, 5G). Oesophagus divided into anterior muscular and much longer, somewhat wider posterior portion (Fig. 3A); length ratio of portions 1:6.8–9.7. Nerve ring encircles muscular oesophagus at level of 4th and 5th rings of spines. Excretory pore situated between 8th and 9th rings of spines in male, and between 9th and 10th rings in female (Figs. 3A, 3B, 4A, 4H, 4I, 5B, 5C, 7B). Males smaller than gravid females.

**Figure 6:**
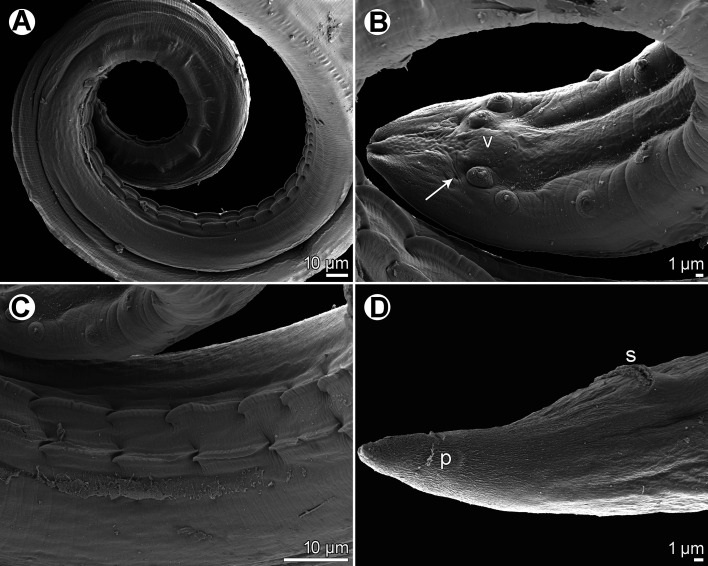
*Spinitectus micracanthus* Christian, 1972 from *Lepomis macrochirus*, Texas, scanning electron micrographs. (A) Posterior end of male with distinct ventral longitudinal precloacal cuticular ridges, lateral view; (B) posterior part of male tail, ventrolateral view (arrow indicates phasmid); (C) precloacal cuticular ridges, ventral view; (D) female tail, lateral view. (p) phasmid; (s) anus; (v) median caudal protuberance.

**Figure 7:**
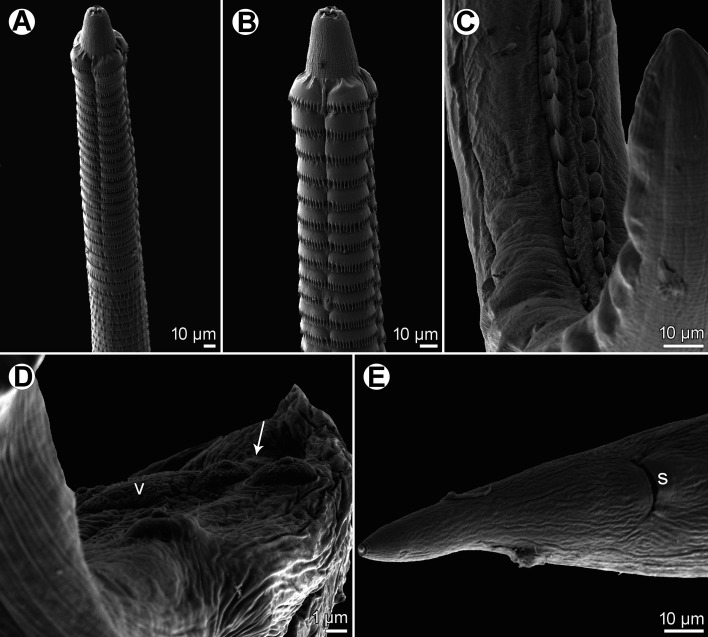
*Spinitectus micracanthus* Christian, 1972 from *Micropterus salmoides*, South Carolina, scanning electron micrographs. (A) Anterior end of female, ventral view; (B) same, larger magnification; (C) ventral precloacal cuticular ridges, ventral view; (D) posterior end of male tail with two posteriormost pairs of postanal papillae, ventrolateral view (arrow indicates phasmid); (E) female tail, ventral view. (s) Anus; (v) median caudal protuberance.

*Male* (5 specimens from *L*. *macrochirus*, Texas; measurements of 5 specimens from *M*. *salmoides*, South Carolina, in parentheses): Length of body 3.81–5.15 (4.19–4.83) mm, maximum width 63–99 (75–99). First ring of spines 57–75 (66–87) from anterior extremity; spines in this ring 3 (3) long. Maximum length of spines 3–6 (3–5). Length of vestibule including prostom 75–87 (87–90); prostom 9–15 (9–12) long, 9–12 (9–12) wide. Muscular oesophagus 186–219 (186–243) long, 18–21 (18–21) wide; glandular oesophagus 1.28–1.78 (1.62–2.12) mm long, 36–54 (45–60) wide; length ratio of both parts of oesophagus 1:6.8–9.1 (1:7.9–8.8). Length of entire oesophagus and vestibule represents 37–46% (44–51%) of body length. Nerve ring and excretory pore 120–123 (120–135) and 171–216 (192–216), respectively, from anterior extremity. Posterior end of body ventrally curved, provided with well-developed vesiculated subventral alae. Ventral precloacal ridges (area rugosa) present, formed by 2 longitudinal rows of tessellated outgrowths (Figs. 6A, 6C, 7C). Preanal papillae: 4 equally spaced pairs of subventral pedunculate papillae. Postanal papillae: 6 pairs of subventral pedunculate papillae of which papillae of last pair shifted more ventrally; small ventral protuberance present between postanal papillae of last pair (Figs. 3L, 3M, 5I, 6B, 7D). Phasmids small, situated just posterior to papillae of last pair (Figs. 3M, 5I, 6B, 7D). Large (left) spicule narrow, 213–233 (233–276) long, with pointed tip; length of its shaft 75–84 (84–105), *i.e.*, 32–38% (34–40%) of spicule length (Figs. 3I, 3L). Small (right) spicule narrow, boat-shaped, 69–87 (81–90) long (Figs. 3J, 3L). Length ratio of spicules 1:2.5–3.2 (1:2.8–3.1). Tail conical, 96–129 (90–123) long, with minute cuticular spike at tip (Figs. 3L, 3M, 5F, 5I, 6D, 7B).

*Female* (5 gravid specimens from *L*. *macrochirus*, Texas; measurements of 3 gravid specimens from *M*. *salmoides*, South Carolina, in parentheses): Length of body 7.40–10.01 (7.03–10.00) mm, maximum width 96–150 (120–138). First ring of spines 63–75 (72–99) from anterior extremity; largest spines 3–6 (3–5) long. Length of vestibule including prostom 75–87 (87–102); funnel-shaped prostom 12 (9–12) long, 12 (9–12) wide. Muscular oesophagus 207–264 (192–240) long, maximum width 21–39 (21); glandular oesophagus 1.26–2.30 (1.86–2.33) mm long, 45–78 (63–72) wide; length ratio of both parts of oesophagus 1:7.6–9.2 (1:9.7). Length of entire oesophagus and vestibule represents 23–30% (26–31%) of body length. Nerve ring and excretory pore 117–132 (117–147) and 168–207 (195–233), respectively from anterior extremity. Vulva situated in posterior half of body, 4.33–5.81 (4.26–4.96) mm from anterior extremity, *i.e.*, at 51–58% (55–61%) of body length; vulval lips not elevated. Vagina muscular, short, directed posteriorly from vulva (Fig. 3G). Fully developed eggs in uterus oval, thick-walled, with smooth surface, each containing larva (Fig. 3H); size 36–42 × 24–27 (39–42 × 24–27); thickness of shell 5–6 (3). No egg filaments or superficial swellings present. Tail conical, smooth (without minute spines), 93–111 (117–126) long; pair of small lateral phasmids situated at short distance from posterior extremity (Figs. 3K, 6D, 7E).

*Female fourth-stage larva* (1 specimen from *L*. *macrochirus*, Texas): Body length 2.27 mm, maximum width 54. Cuticle with numerous rings of spines; first ring of spines 57 from anterior extremity. Vestibule 66 long. Length of muscular oesophagus 165, maximum width 15; length of glandular oesophagus 898, maximum width 30; length ratio of both parts of oesophagus 1:5.4. Entire oesophagus and vestibule represent 45% of body length. Nerve ring 96 from anterior extremity; deirids and excretory pore not located. Vulva situated in posterior half of body, 1.71 mm from anterior extremity, *i.e.*, at 64% of body length.

#### Remarks

*Spinitectus micracanthus* was described by Christian [11] from the intestine of *Lepomis macrochirus* in Ohio, based solely on LM examination of specimens. Christian contrasted the Ohio collection with the two then known congeneric species parasitising North American freshwater fishes, *S*. *gracilis* and *S*. *carolini*, as redescribed by Mueller & Van Cleave [43]. The description differentiated the species from the more similar *S*. *carolini* mainly by 1) the body length of gravid females (16–20 mm *vs.* 7–8 mm), 2) the presence of 56–72 small, short spines per ring (*vs.* 25–35 very long spines per ring), 3) two (*vs.* allegedly four–five?) ventral precloacal ridges (although reported as “a series of rows” by Mueller & Van Cleave [43]), and 4) and the location of the excretory pore between the 7th and 8th rings of spines in males and between the 9th and 10th rings in females (*vs* between 8th and 9th rings).

However, the difference in the body lengths between *S*. *micracanthus* and *S*. *carolini* noted by Christian [11] can be questioned, because the body lengths of adult males and gravid females in *Spinitectus* may nearly double in length over time, as documented for *S*. *inermis* (Zeder, 1800), a parasite of eels, *Anguilla anguilla* (Linnaeus) in Europe [29, 30, 34].

The location of the excretory pore in relation to rings of spines is one of the most important taxonomic features in *Spinitectus* [29]. However, this structure is often difficult to locate using solely LM; but in contrast, it is usually readily visible in SEM micrographs. Therefore, the assertion by Christian [11] (based solely on LM) that the position of the excretory pore in males of *S*. *micracanthus* is between the 7th and 8th rings of spines may very well be incorrect. Indeed, SEM imagery from the present study reveals that the excretory pore of *S*. *micracanthus* is consistently located between the 8th and 9th rings in the male (also illustrated by Mueller and Van Cleave [43] for *S*. *carolini*) and between the 9th and 10th rings in the female (also illustrated by Christian [11]). Consequently, the location of the excretory pore of *S*. *micracanthus* is apparently identical with that of *S*. *carolini*. Therefore, the main distinction between *S*. *micracanthus* and *S*. *carolini* is the number of cuticular spines per ring and their smaller size in the former species.

The morphometrics of available *Spinitectus* specimens from centrarchids in the present material are more or less in agreement with those in the original description of *S*. *micracanthus* and thus, these specimens are considered to belong to this species. Jilek & Crites [23] were the first to examine *S*. *micracanthus* by SEM, but only details of the mouth structure, body spination, deirids and the egg surface were reported. The present detailed LM and SEM examination of this species has 1) determined the actual location of the excretory pore in both males and females, 2) determined the exact structure of the male tail tip, 3) for the first time revealed the presence of sublabia, phasmids and the median ventral protuberance at the level of the last pair of postanal papillae, and 4) confirmed the shape and location of deirids and the presence of two longitudinal ventral precloacal ridges.

Apparently, the main definitive hosts (as defined by Odening [44]) of *S*. *micracanthus* are fishes of the family Centrarchidae, particularly those of the genera *Lepomis* Rafinesque and *Micropterus* Lacepède, whereas fishes of most other host families probably serve as facultative hosts, such as paratenic, paradefinitive or postcyclic hosts. In addition to Christian’s data [11], from Ohio, *S*. *micracanthus* was subsequently reported in the USA from its type host, *L*. *macrochirus*, *e.g.* by Keppner [25] in Missouri, Jilek & Crites [20] in Ohio and Underwood & Dronen [49] in Texas; also the *Spinitectus* from *L*. *macrochirus* in Louisiana [48] and those in Texas from *Gambusia geiseri* and *G*. *affinis* [14, 15], which the authors had identified as *S*. *carolini*, probably also belonged to *S*. *micracanthus*.

The life cycle of *S*. *micracanthus* was studied in detail by Keppner [25], who had found ephemeropteran nymphs of *Hexagenia* sp. to be experimentally suitable intermediate hosts.

Jilek & Crites [21–23] provided experimental data on the development of the related species *S*. *carolini*; they also found that the larvae of various aquatic insects from several orders can experimentally serve as suitable intermediate hosts: Ephemeroptera (*Baetis* Leach, *Caenis* Stephens, *Ephemerella* Welsh, *Heptagenia* Welsh, *Hexagenia* Welsh, *Stenonema* Traver), Odonata (*Gomphus* Leach, *Pachydiplax* Brauer), Plecoptera (*Ischnura* Chaperpentier, *Neoperla* Needham) and Diptera (*Chironomus* Meigen) [22]. Probably *S*. *micracanthus* has a similar range of possible intermediate hosts.

## Discussion

In the vast area of North America, the first species of *Spinitectus* was described by Linton [28] as *Filaria serrata* Linton, 1901 from the white hake *Urophycis tenuis* (Mitchill) (Phycidae, Gadiformes) from off the Atlantic coast of the USA. However, its description was inadequate, with an evident mistake in the given lengths of the spicules [3]. Later Railliet & Henry [46] assigned Linton’s nematodes to *Spinitectus* and proposed for them a new name, *Spinitectus cristatus*. However, according to the International Code of Zoological Nomenclature [19] (Article 11.6.1), the name *S*. *cristatus* is unavailable and should be considered a junior synonym of *S*. *serratus* (Linton, 1901) n. comb. Nevertheless, *S*. *cristatus* Railliet & Henry, 1915 was reported by Rees [47] in *Molva molva* (Linnaeus) (Lotidae, Gadiformes) from the Porcupine Bank, North–East Atlantic, but Berland [3] questioned this finding, and Køie [27] considered Rees’s nematodes as possibly identical with *Spinitectus oviflagellis* Fourment, 1884. The latter species (*S*. *oviflagellis*), redescribed in detail by Moravec & Klimpel [39] from specimens in *Macrourus berlax* Lacepède (Macrouridae, Gadiformes) of the eastern Greenland Sea, is a parasite mainly of gadiform fishes and it is frequently reported from off the Atlantic coast of Canada and the USA [1, 26]. Taking into account the report that the definitive host of *S*. *serratus* is a gadiform fish, both names *S*. *serratus* and *S*. *cristatus* should be considered junior synonyms of *S*. *oviflagellis*.

Ward & Magath [50] were the first to describe a species of *Spinitectus* from North American freshwater fishes, which included *Pomoxis nigromaculatus* (Lesueur) (Centrarchidae), *Aplodinotus grunniens* Rafinesque (Sciaenidae) and *Morone chrysops* (Rafinesque) (Moronidae). However, their description of *S*. *gracilis* Ward & Magath, 1917, which was based on specimens from the Mississippi River in Iowa, is very incomplete and also erroneous (the authors even wrote that the male had no genital papillae). The authors provided no illustrations of *S*. *gracilis*, and it is even possible that their collections represented two or more species. Later, Holl [18] described the second congeneric species, *Spinitectus carolini* Holl, 1928, in North American freshwater fishes, from *Lepomis gibbosus* (Linnaeus) and *L*. *gulosus* (Cuvier) (Centrarchidae) in North Carolina, USA, which he had distinguished from the morphologically similar *S*. *gracilis* by the presence of male papillae. Nevertheless, due to inadequate descriptions of *S*. *gracilis* and *S*. *carolini*, both of these species were apparently confused by subsequent North American authors in their mostly faunistic-survey papers.

While studying helminth parasites of fishes from Oneida Lake, state of New York, Mueller & Van Cleave [43] attempted to revise and redescribe *S*. *gracilis* and *S*. *carolini* in order that they might identify the numerous specimens of *Spinitectus* found in fishes of this lake. They provided a relatively good redescription of *S*. *gracilis* and found that the conspicuously short vestibule and the position of the excretory pore at the level of the 4th ring of cuticular spines were the main characters of this species. They reported a total of nine fish species from four orders and four families that were serving as hosts for *S*. *gracilis* in Oneida Lake, including Centrarchiformes: Centrarchidae (three species), Esociformes: Esocidae (two species), Gadiformes: Lotidae (one species) and Salmoniformes: Salmonidae (two species). All the other available specimens of *Spinitectus* from Oneida Lake with a long vestibule extending posteriorly to the anterior rings of cuticular spines, Mueller & Van Cleave [43] identified as *S*. *carolini*, having taken into account the alleged presence of a small ventral hook on the tip of the small (right) spicule. Although such a small ventral hook was illustrated in the original description of *S*. *carolini* by Holl [18], it is quite likely that a strongly sclerotised distal tip of this boat-shaped spicule with a short part of the less-sclerotised spicule lateral extension was erroneously considered to be a hook. The small (right) spicule of *Spinitectus* and some related nematodes (*e.g.*, *Rhabdochona* spp.) serves like a gubernaculum on the ventral surface of which the long (left) spicule moves, and the presumptive ventral hook would interfere with this movement. Moreover, the small spicule observed in different positions may seem to have somewhat different shapes, and this could have caused the perceived misinterpretations.

The illustration of Holl [18] of the anterior end of *S*. *carolini* (Holl’s fig. 1) clearly shows that the vestibules of these specimens are very short, representing about half the distance between the anterior end of the oesophagus and the anterior extremity. This discrepancy in the lengths of the vestibule (short in *S*. *gracilis* and long in specimens identified as *S*. *carolini*) in the paper of Holl [18] was explained in Mueller & Van Cleave [43] by suggesting that Holl’s collection used for the description probably also included specimens of *S*. *gracilis*.

Since the presence/absence of the minute ventral hook on the tip of the small spicule appeared to be a feature of doubtful taxonomic utility (see above), whereas the very short vestibule typical of S. *gracilis* seemed to separate this species explicitly from the specimens later identified by Mueller & Van Cleave [43] as *S*. *carolini*, we were originally inclined to consider *S*. *carolini* a junior synonym of *S*. *gracilis*. Because Mueller & Van Cleave [43] did not examine Holl’s specimens, we assumed they were no longer available. However, Prof. A. Choudhury has recently informed us that he found the type specimens of *S*. *carolini* deposited in the Smithsonian National Museum of Natural History, Washington consisting of the male “type” (= holotype) from the intestine of *L*. *gulosus* and the female paratype from the intestine of *L*. *gibbosus*. According to him, the vestibule of the holotype is of the ‘long’ type and ends at the first transverse ring of spines and there are 7–8 spines per sector (*i.e.*, 28–32 per ring) in the first two rings. This shows that *S*. *carolini* is a valid species, different from *S*. *gracilis*.

*Spinitectus carolini* was relatively well redescribed by Mueller & Van Cleave [43] from specimens collected from three species of centrarchid fishes (*Ambloplites rupestris* (Rafinesque), *Lepomis gibbosus* and *Micropterus dolomieu* Lacepède) in Oneida Lake. The authors refer to the presence of “about 25–35 very long spines per ring” in their *S*. *carolini* redescription, but their fig. 31 shows only short spines similar to those illustrated in their fig. 32 for *S*. *gracilis*. Since the observations of these authors were based solely on LM and they did not study the anterior ends of specimens in apical view, their data need not be considered quite exact. Regarding the wide range of hosts reported for *S*. *gracilis* and *S*. *carolini* in Oneida Lake, it may well be that more than two species of *Spinitectus* were included in that collection, because some *Spinitectus* spp. parasitising North American freshwater fishes were not known at that time. For example, while Mueller & Van Cleave [43] listed ictalurids as hosts of *S*. *gracilis* in Oneida Lake, another species, *S*. *tabascoensis* Moravec, García-Magaña & Salgado-Maldonado, 2002 (syn. *S*. *macrospinosus* Choudhury & Perryman, 2003), characterised by very long spines, was described from North American ictalurid catfishes in 2002 [10, 37].

The identification of *Spinitectus* specimens characterised by the presence of a long vestibule and with the excretory pore between the 8th and 9th rings of spines as *S*. *carolini* by Mueller & Van Cleave [43] has been followed by all subsequent authors (*e.g.*, [1, 10, 11, 23, 24]). Jilek & Crites [23] published a SEM study comparing the cuticular spines and mouth structures of *S*. *carolini* (sensu Mueller & Van Cleave [43]), *S*. *gracilis*, *S*. *micracanthus* and *S*. *beaveri* Overstreet, 1970. However, those authors disregarded the location of the excretory pore in their specimens and their data on the number (20–30) of cuticular spines per ring in *S*. *carolini* need not be regarded as correct, because specimens were not studied in apical view. Moreover, Jilek & Crites [24] demonstrated experimentally that *S*. *carolini*, originally obtained from the centrarchid *Ambloplites rupestris*, can complete its life cycle and development in *Lepomis macrochirus*, the type and most frequent definitive host of *S*. *micracanthus*. Uncertainty in distinguishing between *S*. *carolini* and *S*. *micracanthus* was confirmed, for example, when Underwood & Dronen [49] reported that the specimens found in fishes of the Upper San Marcos River, Texas represented a mixture of *S*. *carolini* and *S*. *micracanthus* and were considered together; they recorded the allegedly mixed *S*. *carolini*/*micracanthus* infections in the intestine of 11 fish species (nine centrarchids, one ictalurid and one characid), whereas we found only *S*. *micracanthus* intestinal infections in five species of centrarchids (also reported by Underwood & Dronen [49]) in the same locality (see above).

In contrast to the broad host latitude recorded by Mueller & Van Cleave [43] for nematodes identified as *S*. *gracilis* and *S*. *carolini*, which are reported from many host species belonging to multiple fish families and orders and even from some amphibians [17], a certain degree of host specificity has been observed for *Spinitectus* spp. in other geographical regions such as, *e.g.*, Africa or South America [31, 35]. However, what appears to be a broad host latitude of the two above-mentioned species may be due to wrong assignment of specimens to species due to failure to recognise that different facultative host categories (*e.g.*, paratenic, paradefinitive or postcyclic hosts) had been considered to be the definitive hosts of some collections. For instance, the definitive hosts of *S*. *micracanthus* in the present material from the Upper San Marcos River, Texas were found to be centrarchids (*Lepomis*, *Micropterus*), but the recorded hosts of conspecific nematode larvae in the same locality, the characid *A*. *mexicanus* and the poeciliid *G*. *geiseri*, should be considered paratenic hosts, in which the *Spinitectus* larvae, having been acquired *via* ingestion of infected insects, are unable to mature. Subsequent ingestion of these paratenic fish hosts by definitive hosts may then provide a secondary route to complete the life cycle.

It now appears that North American centrarchids serve as the main definitive hosts of *S*. *carolini*, *S*. *gracilis* and *S*. *micracanthus*. According to Underwood & Dronen [49], *S*. *gracilis* occurs in the stomach of these definitive hosts, whereas *S*. *carolini* and *S*. *micracanthus* infect the intestine. However, it is likely that the juvenile specimens of *S*. *gracilis* may also be found in the intestine, as well as adult specimens of the same species in facultative postcyclic hosts. It is interesting that *S*. *acipenseri*, which is morphologically similar to *S*. *gracilis*, also occurs in the stomach of its definitive host (sturgeon).

At present, a total of 13 valid species of *Spinitectus* are known to occur in North American waters, eleven parasitising freshwater fishes and two parasitising marine fishes. While all species from freshwater fishes and one from marine fishes belong to the nominotypical subgenus *Spinitectus* Fourment, 1884, one species from marine fishes belongs to the subgenus *Paraspinitectus* Moravec & Justine, 2009, a subgenus created by Moravec & Justine [38] for species of *Spinitectus* having markedly reduced pseudolabia without inner extensions, with the type species for *Paraspinitectus* being *S*. (*P*.) *beaveri* Overstreet, 1970.

The following valid species of *Spinitectus* have been reported from freshwater fishes in North America: *S*. *acipenseri*, *S*. *agonostomi* Moravec & Baruš, 1971, *S*. *carolini*, *S*. *gracilis*, *S*. *humbertoi* Caspeta-Mandujano & Moravec, 2000, *S*. *mariaisabelae* Caspeta-Mandujano, Cabañas-Carranza & Salgado-Maldonado, 2007, *S*. *mexicanus* Caspeta-Mandujano, Moravec & Salgado-Maldonado, 2000, *S*. *micracanthus*, *S*. *mixtecoensis* Barrios-Gutiérrez, Santacruz, Martínez-Ramírez, Rubio-Godoy & Oinacho-Pinacho, 2019, *S*. *osorioi* Choudhury & Pérez-Ponce de León, 2001 and *S*. *tabascoensis* (syn. *S*. *macrospinosus*) (see, *e.g.*, [2, 4–7, 9–11, 36, 42, 50]). Based on molecular methods, Choudhury & Nadler [8] resurrected *S*. *macrospinosus* Choudhury & Perryman, 2003 as a valid species, but this is not supported by morphological or ecological differences from *S*. *tabascoensis*; so, for the time being, we still consider both forms as synonymous. The species of *Spinitectus* from marine fishes in North American waters are represented only by *S*. *beaveri* and *S*. *oviflagellis* (syn. *S*. *serratus*) [1, 28, 39, 45]. Other *Spinitectus*-like nematodes recorded from North American marine fishes are *Ctenascarophis lesteri* Crites, Overstreet & Maung, 1993 and *Prospinitectus exiguus* Crites, Overstreet & Maung, 1993 [12].

It is necessary to emphasize that further studies of *Spinitectus* species parasitising North American fishes are essential before subsequent researchers can properly diagnose the fauna of these nematodes, their host-parasite relationships and biology, and especially their life cycles. Very important will be molecular studies of individual species of *Spinitectus*. Unfortunately, only six nominal species, *S*. *carolini*, *S*. *gracilis*, *S*. *humbertoi*, *S*. *macrospinosus* (= syn. of *S*. *tabascoensis*), *S*. *mixtecoensis* and *S*. *tabascoensis* have so far been sequenced [2, 8, 13].

It is also important to understand that many of the taxonomic problems revealed in this paper were caused by exclusive reliance on LM as the anatomical investigative tool. Most of the problems we have solved herein were solved *via* SEM studies, and the solution of the remaining mysteries should not be attempted without SEM work to supplement LM studies.

### Key to species of *Spinitectus* from North American freshwater fishes:

1 Vestibule very short, its posterior end far anterior to 1st ring of cuticular spines. Excretory pore near 4th ring of spines. Vagina directed anteriad from vulva. Anterior rings of spines interrupted by 2 lateral narrow spineless longitudinal lanes ……………………………………………………………………………………. 2

– Vestibule long, reaching posteriad to 1st or 2nd ring of cuticular spines. Excretory pore posterior to 5th ring of spines. Vagina directed posteriad from vulva. Anterior rings of spines interrupted by 4 (1 dorsal, 1 ventral and 2 lateral) narrow spineless longitudinal lanes …………………………………………………….………..…. 3

2 Number of spines in 1st ring 20–22. Posterior end of small spicule with large dorsal barb. Body length of gravid female less than 9 mm. Female tail with minute isolated spines. Vulva markedly elevated. Parasitic in Acipenseridae (*Acipenser fulvescens*); Central Canada (Manitoba, Ontario, Saskatchewan) …. ***S*. *acipenseri***

– Number of spines in 1st ring 35–50. Posterior end of small spicule with small dorsal barb. Gravid female 10–19 mm long. Female tail smooth, without spines. Vulva not elevated. Parasitic allegedly in many fish species of different families; USA, Canada ………………………….…….……………………….. ***S*. *gracilis***

3 Left spicule 0.50–1.32 mm long. Eggs smooth or with lateral swellings ….….. 4

– Left spicule less than 500 μm long. Eggs smooth ……………………..……… 5

4 Excretory pore just posterior to 5th ring of spines. Number of spines in 1st ring 30–50. Left spicule 0.50–1.32 mm long. Eggs with lateral swellings. Size of eggs 36 × 30 μm. Female tail smooth, without spines. Parasitic in Mugilidae (*Agonostomus monticola*) (in Mexico reported also from some other fishes); Cuba, Guadeloupe, Puerto Rico, Mexico (Veracruz, Jalisco) ………….. ***S*. *agonostomi***

– Excretory pore between 7th and 8th rings of spines. Number of spines in 1st ring 52–60. Left spicule 1.00–1.10 mm long. Eggs smooth, without lateral swellings. Size of eggs 57–59 × 38–39 μm. Female tail with minute spines. Parasitic in Cyprinodontidae (*Profundulus punctatus*); Mexico (Chiapas) … ***S*. *mariaisabelae***

5 Excretory pore between 8th and 9th rings of spines in male and between 9th and 10th rings in female ………………………..…………………………………… 6

– Excretory pore situated more anteriorly. ……………………………………….. 7

6 Gravid female about 7–8 mm long. Number of spines in 1st ring 20–35. Length of left spicule about 275 μm. Reported mainly from Centrarchidae (*Ambloplites rupestris*, *Lepomis* spp., *Micropterus dolomieu*); USA …………..…. ***S*. *carolini***

– Gravid female 16–20 mm long. Number of spines in 1st ring 56–72. Length of left spicule 296–312 μm. Parasitic mainly in Centrarchidae (*Lepomis* spp., *Micropterus salmoides*); USA (Ohio, Texas, S. Carolina) …………….……. ***S*. *micracanthus***

7 Excretory pore between 5th and 6th rings of spines. Number of spines in 1st ring 12–20; spines not markedly long. Number of large spines in sectors of rings diminishing posteriorly so that sectors of last rings with large spines are formed by a single spine each. Female tail with minute spines. Parasitic in Poeciliidae (*Heterandria bimaculata*); Mexico (Veracruz) …………………… ***S*. *mexicanus***

– Excretory pore posterior to level of 6th ring of spines ………………….…….... 8

8 Excretory pore between 6th and 7th rings of spines …………………………….. 9

– Excretory pore between 7th and 8th rings of spines ……………………………. 10

9 Number of spines in 1st ring 18–22; anterior rings of large spines numerous (17 or more), arranged in 4 inconspicuously separated sectors; spines markedly long. Numbers of large spines not diminishing posteriorly. Female tail smooth, without spines. Parasitic in Ictaluridae (*Ictalurus furcatus*, *I*. *punctatus*); Mexico (Tabasco, Chiapas), USA (Kentucky-Tennessee, Oklahoma), Canada (Manitoba) .……………………………………………………………..…… ***S*. *tabascoensis***

– Number of spines in 1st ring 28–44; spines not markedly long. Female tail with terminal mucron with many minute processes. Parasitic in Atherinopsidae (*Atherinella*, *Chirostoma*) and Gerreidae (*Eugerres*); Mexico (Chiapas, Michoacán) ……………………………………………………...…… ***S*. *osorioi***

10 Number of spines in 1st ring 22–28; 7 anterior rings of large spines present, each ring being divided into 4 conspicuously separated sectors of spines; spines not markedly long. Female tail with minute spines. Parasitic in Profundulidae (*Profundulus punctatus*); Mexico (Oaxaca) ………………….… ***S*. *mixtecoensis***

– Number of spines in 1st ring 36–38. Female tail with minute spines, without terminal appendage. Parasitic in Profundulidae (*Profundulus labialis*); Mexico (Guerrero) ………………………………………………………… ***S*. *humbertoi***

### Key to *Spinitectus*-like nematodes parasitic in North American marine fishes:

1 Body surface armed with 4 sublateral longitudinal rows of raised transverse combs, each bearing 3 or more posteriorly directed spines. Combs with spines begin anteriorly just posterior to level of muscular and glandular oesophageal junction. Lateral alae present. Vestibule long. Eggs with several filaments at both poles. Stomach parasites of *Katsuwonus pelamis*; Puerto Rico (also in Pacific region outside N. America) ………………………..…… ***Ctenascarophis lesteri***

– Body surface armed with transverse rings of cuticular spines; rings uninterrupted or interrupted by 2 or 4 narrow spineless longitudinal lanes. First ring of spines short distance posterior to anterior extremity. Lateral alae absent. Vestibule short, long or absent. Eggs with or without filaments ……………………………………… 2

2 Vestibule absent. Pseudolabia reduced, each with well-developed pointed apical tooth. Eggs smooth, without filaments. Parasitic in digestive tract of *Katsuwonus pelamis*; Puerto Rico (also in Atlantic and Pacific regions outside N. America) …………………. ……………………………………….***Prospinitectus exiguus***

– Vestibule present. Pseudolabia well developed or reduced, without apical teeth …………………………………………………………………………………… 3

3 Pseudolabia well developed, with inner extensions (subg. *Spinitectus*). Eggs with polar filaments. Rings of spines begin at about mid-length of vestibule. Excretory pore between 8th and 9th rings of spines. Parasitic in digestive tract of Gadiformes (*e.g.*, *Coryphaenoides*, *Gaidropsarus*, *Macrourus*, *Merlangius*, *Molva*, *Urophycis*) in North Atlantic ………………………………..………. ***Spinitectus oviflagellis***

– Pseudolabia markedly reduced, without inner extensions (subg. *Paraspinitectus*). Eggs without filaments. First ring of spines just posterior to prostom. Excretory pore probably between 14th and 15th rings of spines. Parasitic in stomach of Albuliformes (*Albula*) off Florida, North Atlantic ..............… ***Spinitectus beaveri***
